# The Effect of Breakfast Skipping and Late Night Eating on Body Mass Index and Glycemic Control Among Patients With Type 2 Diabetes Mellitus

**DOI:** 10.7759/cureus.15853

**Published:** 2021-06-23

**Authors:** Hyder Mirghani

**Affiliations:** 1 Internal Medicine, University of Tabuk, Tabuk, SAU

**Keywords:** breakfast skipping, late-night eating, hba1c, obesity, saudi arabia

## Abstract

Introduction

There is an increasing awareness regarding the effects of chrono-nutrition on glycemic control and weight regulation. Therefore, this study aimed to determine the relationship between breakfast skipping and late-night eating to body mass index and glycemic control among patients with type 2 diabetes.

Subjects and methods

This cross-sectional study was conducted among 310 patients with diabetes in Tabuk City, Saudi Arabia during the period from December 2020 to April 2021. A structured questionnaire was used to interview the participants, the following were reported: demographic data, breakfast skipping, late-night eating, smoking, level of exercise, family history of diabetes, and diabetes complications. Weight and height were measured to calculate the body mass index (BMI), and the last glycated hemoglobin was collected to estimate the degree of glycemic control. Statistical Package for Social Sciences (SPSS Statistics, IBM Corp., Armonk, USA) was used for data analysis. The ethical committee of the University of Tabuk approved the research.

Results

Out of 310 patients with diabetes (54.8% women), nearly half (45.2%) were breakfast-skippers and 20% eat late at night. Breakfast skipping was correlated with BMI (Wald, 5.481, *95% *CI, 0.154-0.847, P-value, 0.019). A positive direct correlation was evident between late dinner intake, BMI, and HbA1c (Wald, 4.210, 95% CI*,* 0.743-0.993, P-value, 0.04 for HbA1c, and Wald, 6.777, 95% CI*,* 1.0221-1.165*, *P-value, 0.009 for BMI).

Conclusion

Breakfast skipping and late dinner intake were associated with obesity, while only late dinner consumption was associated with poor glycemic control. Further larger multi-center studies investigating the chronotype and glycemic index are recommended.

## Introduction

Diabetes mellitus is a global health burden and the number is increasing at an alarming rate. Currently, 285 million are affected by this lifelong morbid metabolic disorder. The number is projected to reach 438 million by the year 2030 and the Kingdom of Saudi Arabia is among the countries with the highest prevalence, according to International Diabetes Federation [1, 2].

Lifestyle management is a fundamental aspect of diabetes care from the initial evaluation through regular follow-up, assessment for complications, and subsequent management. Nutrition therapy plays an integral role for every patient with diabetes mellitus, and every diabetic patient should receive individualized nutritional therapy. The American Diabetes Association recommends the glycated hemoglobin (HbA1c) be targeted to <7 to prevent or delay the microvascular complications. For patients with type 2 diabetes mellitus, medical nutrition therapy is associated with 0.5-2% glycated hemoglobin reduction [3,4].

Breakfast skipping among adults is associated with insulin resistance, hypertension, elevated lipids concentration, and increasing body weight. Eating late at night (late dinner) regardless of meal composition had been linked to higher cardiovascular disease risk [5]. On the other hand, a recent review indicated that the consumption of whole grains and cereal fiber in breakfast and limiting rapidly available carbohydrates lowers insulinemia and glycemia [6].

Many factors have been suggested to link type 2 diabetes mellitus and breakfast skipping including nocturnal activity, decreased appetite, smoking, and lack of exercise, but the mechanisms are poorly understood. Furthermore, the habit of breakfast skipping could be associated with late dinner owing to various factors including fatigue, poor appetite, and lack of time [7].

The meal timing and the daily rhythm of feeding-fasting are emerging as important health determinants. Previous literature observed that decreased length of overnight fasting or increased late-night eating enhances the risk of metabolic diseases like diabetes and obesity. The daily eating pattern is a possible significant modifiable aspect of lifestyle to contain metabolic disorders [8]. 

To the best of our knowledge, no researchers have assessed breakfast skipping and dinner timing effects on BMI and HbA1c. Given the above and the fact that glycemic control is an essential element in the prevention of microvascular complications, we conducted this research. In the present study, we aimed to assess breakfast skipping and late dinner intake among patients with type 2 diabetes and their relation to glycemic control.

## Materials and methods

Study type and participants

This cross-sectional study was conducted among 310 diabetic patients in Tabuk City, the Kingdom of Saudi Arabia. A stratified random sampling technique was used to select the participants. The patients were already diagnosed with the diseases according to the American Diabetes Association guidelines.

Inclusion and exclusion criteria

All adults with type 2 diabetes mellitus were included, those with type 1 diabetes and pregnant ladies were excluded from the study. The study was conducted during the period from December 2020 to April 2021.

Sample size

The sample size was calculated using the following formula: n = Z2 P-Q/d2 where Z = 95% confidence (1.96), P = rate of diabetes mellitus in Saudi Arabia [9].

Measures

A structured questionnaire was used to collect the following: age, sex, level of education, the diabetes medications, the diabetes complications, family history of diabetes mellitus, the frequency of breakfast intake, and dinner consumption within the last two hours before sleeping, the level of exercise, and smoking status. The most recent glycated hemoglobin was reported to assess the degree of glycemic control.

The weight and height of all the participants were measured, and the body mass index was calculated using the formula: BMI = Weight in Kg/(Height in meters)2

For this research, the following definitions were adopted:

· Normal weight = BMI 18.5-24.9

· Overweight = 25-29.5

· Obese = 30-40

· Morbid obesity >40

· Breakfast was defined as any food or beverages consumed between 5:00 a.m. and 10:00 a.m. [7]

· Late dinner: eating dinner within two hours before bedtime at least three times per week [10]

· Breakfast skipping: Skipping the breakfast at least three times per week [10]

Participants signed a written informed consent and the ethical committees of the University of Tabuk approved the research (ref. number, UT-155-118-2021).

Statistical analysis

The Statistical Package for Social Sciences (SPSS Statistics version 20, IBM Corp., Armonk, USA) was used for data analysis. Binary logistic regression analysis was used to compare breakfast-skippers and late dinner eaters with non-skippers and early dinner eaters. The data were presented as percentages or mean± SD unless otherwise specified and a P-value of <0.05 was considered significant.

## Results

Among the participants (54.8% women), 77.7% were either obese (50.7%) or overweight (27%), the majority (74.8%) were not reaching glycemic targets, and nearly a half (45.2%) were breakfast-skippers and 20% eat late at night. Regarding diabetes complications, retinopathy was reported in 17.4% while 3.2% and 5.2% had nephropathy and a history of coronary artery disease, respectively. In the current study, 60% reported a family history of diabetes mellitus, 18.8% were cigarette smokers, and 20% were practicing exercise five times/week and for 30 minutes (Table 1).

**Table 1:** Basic Characters of the Study Group

Character	No %
Sex	
Women	170 (54.8%)
Men	140 (45.2%)
BMI	
Obese	154 (50.7%)
Overweight	82 (27%)
Normal	68 (22.4%)
Glycemic control	
Poor (≤7)	332 (74.8%)
Good (>7)	78 (25.2%)
Skipping breakfast	140 (45.2%)
Eating late in the night	62 (20%)
Retinopathy	54 (17.4%)
Nephropathy	10 (3.2%)
History of coronary artery disease	16 (5.2)
Family history of diabetes	186 (60%)
On regular exercise	62 (20%)
Smoking	58 (18.8%)

The current data showed that the mean age was 55.05±9.90 years, the glycated hemoglobin was 8.96±2.47, and the body mass index was 31.01±7.57s. Table 2. 

**Table 2:** Age, BMI, and the Glycated Hemoglobin Among the Study Group

Character	Mean± SD
Age	55.05±9.90
BMI	31.01±7.57
HbA1_c_	8.96±2.47

In the current study, breakfast skipping was correlated with BMI (Wald, 5.481, 95% CI, 0.154-0.847, P-value, 0.019); no correlation was evident between skipping breakfast and late dinner consumption (Wald, 0.009, 95% CI, 0.436-2.488, P-value, 0.927), age (Wald, 0.143, 95% CI, 0. 0.960-1.028, P-value, 0.705), sex (Wald, 0.103, 95% CI, 0.571-2.180, P-value, 0.748), and HbA1c (Wald, 0.518, 95% CI, 0.916-1.209, P-value, 0.472) (Table 3).

**Table 3:** The Relationship Between Breakfasts Skipping, Ages, Sex, Eating Late in the Night, Body Mass Index, HbA1c HbA1c: glycated hemoglobin; df: degrees of freedom

Character	Wald	df	Exp.	95% CI	P-value
Age	0.143	1	0.993	0.960-1.028	0.705
Sex	0.103	1	1.116	0.571-2.180	0.748
BMI	5.481	1	0.361	0.154-0.847	0.019
Eating late in the night	0.009	1	1.042	0.436-2.488	0.927
HbA1c	0.518	1	1.052	0.916-1.209	0.472
Constant	0.436	1	2.215		0.509

Regarding the association of late dinner intake, BMI, and HbA1c, positive direct correlations were observed with a significant statistical difference (Wald, 4.210, 95% CI, 0.743-0.993, P-value, 0.04 for HbA1c, and Wald, 6.777, 95% CI, 1.0221-1.165, P-value, 0.009 for BMI) (Table 4).

**Table 4:** The Relationship Between Eating Late in the Night, Body Mass Index, and HbA1c HbA1c: glycated hemoglobin; df: degrees of freedom

Character	Wald	df	Exp.	95% CI	P-value
HbA1_c_	4.210	1	0.859	0.743-0.993	0.040
BMI	6.777	1	1.091	1.0221-1.165	0.009
constant	5.481	1	1.273		0.845

## Discussion

In the present study, nearly half of patients with diabetes skipped breakfast and one in five ate late in the night. Breakfast skipping and late dinner consumption were associated with obesity. However, a direct significant relationship was found between poor glycemic control and late dinner but not breakfast skipping.

The current study reported a breakfast-skipping rate of 45.2% in line with a previous study published in Japan and reported a prevalence of 39.2% [11]. The current findings are in line with a recent study published in the USA [12]. The association between breakfast skipping and BMI was discussed controversially (some found an association [13] while others found no relationship [14]). In the present survey, breakfast skipping was associated with a high BMI, in agreement with Gouda et al. [15].

The current study showed no significant differences between breakfast skipping and glycated hemoglobin. Similarly, a study published in Japan [7] reported no association between hyperglycemia and breakfast skipping, and the present data were in contradiction to previous studies [7] that concluded an association between breakfast skipping and high glycated hemoglobin. The association between breakfast skipping and diabetes is thought to be mediated by low-grade inflammation and BMI [16]. It is interesting to note that eating late in the night in our study was associated with both higher BMI and glycated hemoglobin in agreement with Nakajima and colleagues who reported similar results [7]. The association of breakfast skipping with obesity and diabetes was observed by previous studies [17, 18]. However, few researchers have assessed both breakfast skipping and late dinner consumption with obesity and poor glycemic control [7, 11]. The effects of late dinner intake on glycemic control are mediated by prolonging the postprandial glucose spike. In addition, insulin sensitivity and glucose tolerance decrease during the night [19]. The high prevalence of poor glycemic control (74.8%) is alarming and supporting previous observations [20]. The current results imply that meal timing is a cheap applicable preventive and therapeutic intervention for weight management and improving glycemic target among patients with diabetes mellitus [21].

The limitations of this study are the small size of the study sample and the fact that the study was conducted at a single diabetes center, so generalization cannot be derived.

## Conclusions

In the current study, a significant direct relationship was found between breakfast skipping, late dinner intake, and body mass index. Late dinner consumption but not breakfast skipping was associated with poor glycemic control. Further, larger multi-center studies focusing on meal amount, chronotype, and glycemic index are highly recommended.

## References

[1] Saeedi P, Petersohn I, Salpea P (2019). Global and regional diabetes prevalence estimates for 2019 and projections for 2030 and 2045: results from the International Diabetes Federation Diabetes Atlas, 9th edition. Diabetes Res Clin Pract.

[2] Khuwaja AK, Lalani S, Dhanani R, Azam IS, Rafique G, White F (2010). Anxiety and depression among outpatients with type 2 diabetes: a multi-centre study of prevalence and associated factors. Diabetol Metab Syndr.

[3] (2017). 4. Lifestyle Management. Diabetes Care.

[4] Coppell KJ, Kataoka M, Williams SM, Chisholm AW, Vorgers SM, Mann JI (2010). Nutritional intervention in patients with type 2 diabetes who are hyperglycaemic despite optimised drug treatment--Lifestyle Over and Above Drugs in Diabetes (LOADD) study: randomised controlled trial. BMJ.

[5] Cahill LE, Chiuve SE, Mekary RA, Jensen MK, Flint AJ, Hu FB, Rimm EB (2013). Prospective study of breakfast eating and incident coronary heart disease in a cohort of male US health professionals. Circulation.

[6] Maki KC, Phillips-Eakley AK, Smith KN (2016). The effects of breakfast consumption and composition on metabolic wellness with a focus on carbohydrate metabolism. Adv Nutr.

[7] Nakajima K, Suwa K (2015). Association of hyperglycemia in a general Japanese population with late-night-dinner eating alone, but not breakfast skipping alone. J Diabetes Metab Disord.

[8] Gupta NJ, Kumar V, Panda S (2017). A camera-phone based study reveals erratic eating pattern and disrupted daily eating-fasting cycle among adults in India. PLoS One.

[9] Alotaibi A, Perry L, Gholizadeh L, Al-Ganmi A (2017). Incidence and prevalence rates of diabetes mellitus in Saudi Arabia: an overview. J Epidemiol Glob Health.

[10] Asao K, Marekani AS, VanCleave J, Rothberg AE (2016). Leptin level and skipping breakfast: the National Health and Nutrition Examination Survey III (NHANES III). Nutrients.

[11] Azami Y, Funakoshi M, Matsumoto H (2019). Long working hours and skipping breakfast concomitant with late evening meals are associated with suboptimal glycemic control among young male Japanese patients with type 2 diabetes. J Diabetes Investig.

[12] Helo D, Appiah L, Bhende KM, Byrd TL, Appiah D (2021). The association of skipping breakfast with cancer-related and all-cause mortality in a national cohort of United States adults. Cancer Causes Control.

[13] Ballon A, Neuenschwander M, Schlesinger S (2019). Breakfast skipping is associated with increased risk of type 2 diabetes among adults: a systematic review and meta-analysis of prospective cohort studies. J Nutr.

[14] Wicherski J, Schlesinger S, Fischer F (2021). Association between breakfast skipping and body weight-a systematic review and meta-analysis of observational longitudinal studies. Nutrients.

[15] Gouda M, Matsukawa M, Iijima H (2018). Associations between eating habits and glycemic control and obesity in Japanese workers with type 2 diabetes mellitus. Diabetes Metab Syndr Obes.

[16] Nas A, Mirza N, Hägele F (2017). Impact of breakfast skipping compared with dinner skipping on regulation of energy balance and metabolic risk. Am J Clin Nutr.

[17] Reutrakul S, Hood MM, Crowley SJ, Morgan MK, Teodori M, Knutson KL (2014). The relationship between breakfast skipping, chronotype, and glycemic control in type 2 diabetes. Chronobiol Int.

[18] Hashimoto Y, Kaji A, Sakai R (2020). Skipping breakfast is associated with glycemic variability in patients with type 2 diabetes. Nutrition.

[19] Sakai R, Hashimoto Y, Ushigome E (2018). Late-night-dinner is associated with poor glycemic control in people with type 2 diabetes: the KAMOGAWA-DM cohort study. Endocr J.

[20] Alzaheb RA, Altemani AH (2018). The prevalence and determinants of poor glycemic control among adults with type 2 diabetes mellitus in Saudi Arabia. Diabetes Metab Syndr Obes.

[21] Beccuti G, Monagheddu C, Evangelista A, Ciccone G, Broglio F, Soldati L, Bo S (2017). Timing of food intake: Sounding the alarm about metabolic impairments? a systematic review. Pharmacol Res.

